# Obituary—Dr. Augustus Woodruff Brown, D.D.S.

**Published:** 1895-08

**Authors:** 


### Obituary.

Died July 5th, at his summer residence in Manchester, Vt., Dr. Augustus Woodruff Brown, D.D.S., age 90. He was born in Litchfield, Conn., and was supposed to be the oldest dentist in America at the time of his death. He practiced in New York City for half a century, and retired in good circumstances, fifteen years ago.

He was at first associated with Dr. Solyman Brown, his eldest brother, whose name is one of the best known in the profession at that period. In their office at 13 Park Place, New York City, the first Dental Society in the world was organized, and the first Dental Journal planned. These were The American Society of Dental Surgeons, of which Dr. Eleazer Parmly was first president, and *The American Journal of Dental Science*, of which Dr. Solyman Brown was the first editor.

Dr. A. W. Brown at one time had the most aristocratic and lucrative practice in New York, and was widely known in social as well as professional circles.

One of the earliest honorary degrees of the Baltimore College of Dental Surgery was conferred upon him. He married Emma Mandeville, who survives him together with two daughters. He had nine children but none of his sons lived to manhood.

Dr. E. Parmly Brown, son of his brother Solyman, studied with him and is the only member of the family now in dentistry.

Funeral services were held at Manchester, and the interment was in the family vault, in old Marble Cemetery, New York City.

